# Before, Between, and After: Enriching Robot Communication Surrounding Collaborative Creative Activities

**DOI:** 10.3389/frobt.2021.662355

**Published:** 2021-04-29

**Authors:** Richard Savery, Lisa Zahray, Gil Weinberg

**Affiliations:** Robotic Musicianship Lab, Georgia Tech Center for Music Technology, Atlanta, GA, United States

**Keywords:** creativity, robotics, music, improvisation, sound, text-to-speech, human-robot interaction

## Abstract

Research in creative robotics continues to expand across all creative domains, including art, music and language. Creative robots are primarily designed to be task specific, with limited research into the implications of their design outside their core task. In the case of a musical robot, this includes when a human sees and interacts with the robot before and after the performance, as well as in between pieces. These non-musical interaction tasks such as the presence of a robot during musical equipment set up, play a key role in the human perception of the robot however have received only limited attention. In this paper, we describe a new audio system using emotional musical prosody, designed to match the creative process of a musical robot for use before, between and after musical performances. Our generation system relies on the creation of a custom dataset for musical prosody. This system is designed foremost to operate in real time and allow rapid generation and dialogue exchange between human and robot. For this reason, the system combines symbolic deep learning through a Conditional Convolution Variational Auto-encoder, with an emotion-tagged audio sampler. We then compare this to a SOTA text-to-speech system in our robotic platform, Shimon the marimba player.We conducted a between-groups study with 100 participants watching a musician interact for 30 s with Shimon. We were able to increase user ratings for the key creativity metrics; novelty and coherence, while maintaining ratings for expressivity across each implementation. Our results also indicated that by communicating in a form that relates to the robot’s core functionality, we can raise likeability and perceived intelligence, while not altering animacy or anthropomorphism. These findings indicate the variation that can occur in the perception of a robot based on interactions surrounding a performance, such as initial meetings and spaces between pieces, in addition to the core creative algorithms.

## 1 Introduction

There is a growing body of work focusing on robots collaborating with humans on creative tasks such as art, language, and music. The development of robotic functionalities leading to and following after collaborative creative tasks has received considerably less attention. These functionalities can address, for example, how a robot communicates and interacts with collaborators between musical improvisations, or before a piece begins or ends. Embodying a creative robot with speech capabilities that do not specifically address its creative capabilities risks distancing collaborators and misrepresenting artistic opportunities. In robotic literature this is referred to as the habitability gap, which addresses the problematic distance between a robot’s implied capabilities and its actual potential output (Moore, 2017). In addition, human-robot collaboration is dependent on the development of a relationship between human and robot (Fischer, 2019). Emotion and personality conveyance has been shown to enhance robotic collaborations, with improved human-robot relationships and increased trust (Bates, 1994). One under-explored approach for an artificial agent to convey emotions is through non-linguistic musical prosody (Savery et al., 2020a). We propose that such an approach could be particularly effective in human-robot collaboration in creative tasks, where emotional expression is at the core of the activity, and where subtle background conveyance of mood can enhance, rather than distract, from the creative activity.

We present a model for generating emotional musical prosody in embedded platforms in real time for creative robots. The system aims to address the habitability gap by enriching human-robot communication before, during and after collaborative creative interaction. To support the system, we have created a new dataset of improvised emotional sung phrases, used to generate new emotional midi phrases through a convolutional variational autoencoder (CVAE) conditioned on emotion.

We implement this system in a marimba playing robot, Shimon, and analyze the impact on users during creativity-based musical interactions. The musical tasks feature call and response musical improvisation over a pre-recorded playback. We compare the perception of common metrics of likeability and perceived intelligence, with the perceived creativity and preferences for interaction as well as Boden’s creativity metrics (Boden, 2009). We demonstrate that by using a creative communication method in addition to the core creative algorithms of a robotic system we are able to improve the interaction based on these metrics. Our implementation leads to the perception of higher levels of creativity in the robot, increased likeability, and improved perceived intelligence.

## 2 Related Work

### 2.1 Human-Robot Communication

Verbal language-based interaction is the prominent form of communication used in human-robot interaction (Mavridis, 2015) covering a wide range of tasks from robot companions (Dautenhahn et al., 2006) to industrial robots (Pires and Azar, 2018). Many robotic interactions do not include language; these non-verbal forms of communication fall into six categories: kinesics, proxemics, haptics, chronemics, vocalics, and presentation (Jones, 2013; Saunderson and Nejat, 2019). Kinesics includes communication through body movement, such as gestures (Gleeson et al., 2013), or facial expressions, while proxemics focuses on the robotic positioning in space, such as the distance from a human collaborator (Walters et al., 2005). Haptics refers to touch based methods (Fukuda et al., 2012), while chronemics includes subtle traits such as hesitation (Moon et al., 2011). Presentation includes the way the robot appears, such as changes based on different behavior (Goetz et al., 2003). The final category, vocalics, includes concepts such as prosody (Crumpton and Bethel, 2016), which have shown to improve trust and other human-robot interaction metrics (Savery et al., 2019a). The vast majority of these communication techniques require significant technical and financial expense and variation to a system, such as adding augmented reality technology or changing robot movements (Saunderson and Nejat, 2019). In comparison, musical prosody can be implemented in an existing system with only minor changes (Savery et al., 2019b).

### 2.2 Musical Generation

Music generation has been widely addressed as a deep learning task (Briot et al., 2017), in particular using LSTMs (Sturm et al., 2016; Wu et al., 2019) and more recently transformers Huang et al. (2018). Music tagged with emotion has also been generated through long short-term memory networks (LSTMs) with logistic regression and used to generate music with sentiment (Ferreira and Whitehead, 2019). Other efforts have used a Biaxial LSTM network (Zhao et al., 2019), generating symbolic polyphonic musical phrases corresponding to Russel’s valence-arousal emotion space (Posner et al., 2005). Variational autoencoders (VAEs) Kingma and Welling (2013); Rezende et al. (2014) use an encoder to represent its input probabilistically in latent space, and a decoder to convert from latent space back to the original input. Such VAEs have seen recent success in music generation tasks, for example, MIDI-VAE which use a VAE with recurrent encoder/decoder pairs to perform style transfer on midi data, changing the genre or composer of a piece (Brunner et al., 2018). MusicVAE employs a hierarchical decoder to better represent the long-term structure present in music, generating midi phrases that were 16 bars (about 30 s) long (Roberts et al., 2018).

## 3 Custom Dataset

For this project we created a custom dataset of 4.22 h of audio recorded by Mary Esther Carter^1^. Carter is a professional vocalist and improviser who the authors have worked with before and were confident would be able to create a dataset matching the projects goals. Before collecting the data, we conducted exploratory sessions with seven different student musicians, comparing their ability to improvise different emotions using different classification systems. We additionally evaluated how well the musicians in this group could recognize the emotions played by other musicians. This process consisted of a 45 min in-person session, with musicians first improvising, followed by an informal interview to discuss the difficulty and their preferences for emotional classifications for improvisation. After these sessions, we decided that the Geneva Emotion Wheel (GEW) (Sacharin et al., 2012) was best suited for our purposes. The GEW is a circular model, containing 20 emotions with emotions and position corresponding to the circumplex model.

Our decision to use the GEW was based on multiple factors, firstly we aimed to capture as large a range of emotions as possible, that could be accurately improvised by musicians in the sessions. In our exploratory study, the GEW balanced between having many recognizable classes, while also avoiding the potential confusion from too many overlapping classes, or the challenge of continuous classes such as the circumplex model. The GEW also has advantages for implementation, with 20 different discrete emotions which can be reduced to four separate classes, aligned with a quadrant from the circumplex model. GEW also includes most of the Eckman’s basic emotions—fear, anger, disgust, sadness, happiness—only leaving out surprise. The ability to potentially reduce our collected dataset between these different models of emotion allows for significant future use cases.

It should be noted that this dataset comes from only one musician, and therefore only captures one perspective on musical emotion. While the dataset can make no claim to represent cross-cultural emotion conveyance and does not create a generalized emotion model, we believe that only collecting data from one person has advantages. By having only one vocalist our system can recreate one person’s emotional style, avoiding incorrectly aggregating multiple styles to remove distinctive individual and stylistic features.

### 3.1 Process and Data

We first created a short list of vocalists who we have worked with in the past. We then conducted Skype calls with three professional vocalists refining the overall plan and describing the process, before asking Mary Carter to record and emotionally label her vocal improvisation. We choose to work with Carter as she had at home access to high quality recording equipment, and the authors have previously worked with her. In the future we expect to record with additional vocalists. Carter was paid $500 to record the samples over a week long period at her home studio, using a template we created in Apple digital audio workstation—Logic Pro, while maintaining the same microphone positioning. For the samples we requested phrases to be between 1 and 20 s, and to spend about 15 min on each emotion, allowing unscripted jumping between any order of the emotions. We allowed deletion of a phrase if the singer felt retroactively that the phrase did not capture the correct emotion. The final recorded dataset includes 2,441 phrases equaling 4.22 h of data with an average of 122 phrases for each emotion. Samples from the dataset can be heard online.^2^

### 3.2 Dataset Validation

**FIGURE 1:**
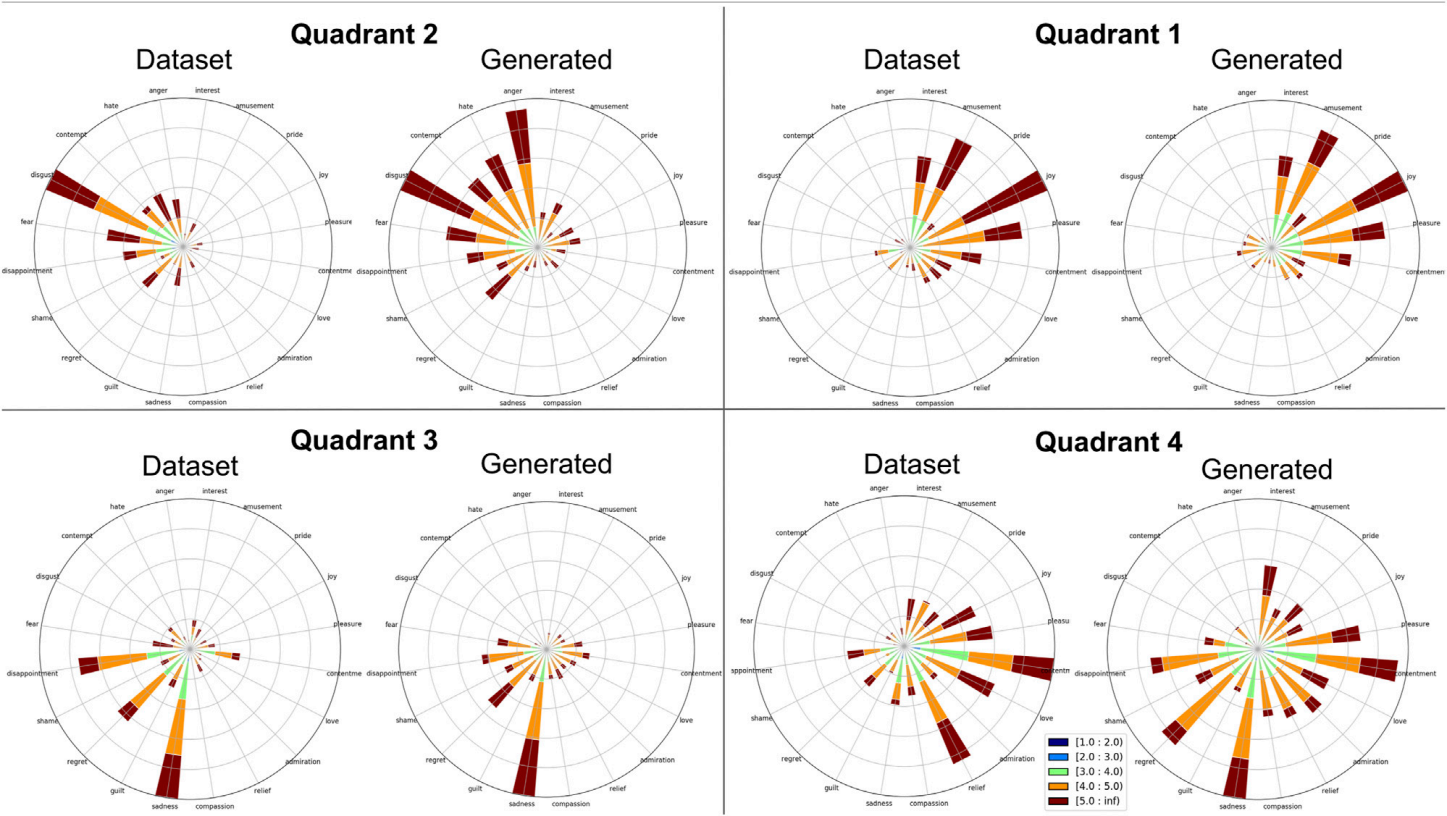
Rose plots of dataset validation and generation evaluation.

To validate the dataset, we performed a study with 45 participants from Prolific and Mechanical Turk, paying each $3. Each question in the survey asked the participant to listen to a phrase and select a location on the wheel corresponding to the emotion and intensity they believed the phrase was trying to convey. Phrases fell under two categories of “best” and “all,” with each participant listening to 60 total phrases selected at random. Between the 45 participants listening to 60 phrases, 2,700 ratings were given, which we believe gave a strong overall rating of the dataset. The “best” category consisted of five phrases for each emotion that were hand-selected by the authors as best representing that emotion. The best emotions were chosen to ensure an even distribution of phrase lengths in each emotion set, with each emotion having a chosen phrase for the lengths, 3, 5, 7, 9, and 11 s. When multiple phrases existed for each length the authors chose phrases that were most distinctive in style from the other emotions, aiming to create a stylistic separation between each emotion class. The “all” category consisted of a phrase sampled from all phrases in the dataset for that emotion, with a new phrase randomly selected for each participant. Rose plots of the validation results that combine the “best” and “all” categories can be seen in Figure 1, separated into each Geneva Wheel quadrant. The plots show strong validation correlation in Quadrants 1, 2 and 3, while Quadrant four showed a higher level of confusion.

### 3.3 Dataset to Midi

We converted each phrase’s audio into a midi representation to use as training data. This process required significant iteration, as we developed a custom pipeline for processing our dataset. This was necessary due to the range of vocal timbre and effect, ranging from clear melodies, to non-pitched effects. We first ran the monophonic pitch detection algorithm CREPE (Kim et al., 2018) on each phrase, which output a frequency and a confidence value for a pitch being present every 0.01 s. As the phrases included breaths and silence, it was necessary to filter out pitches detected with low confidence. We applied a threshold followed by a median filter to the confidence values, and forced each detected pitch region to be at least 0.04 s long.

We next converted the frequencies to midi pitches. We found the most common pitch deviation for each phrase using a histogram of deviations, shifting the midi pitches by this deviation to tune each phrase. We rated onsets timing confidence between 0 and 1. To address glissando, vibrato and other continuous pitch changes, we identified peaks in the absolute value of the pitch derivative, counting an onset only when detecting a pitch for at least 0.04 s.

### 3.4 Scales

Scherer has shown that musical scales—without a melody or rhythm - are able to display emotion (Scherer et al., 2017). We therefore asked the singer to also record scales tagged with emotion to be used in an audio sampler. The audio sampler was designed to play back each note from the recorded scales, in such a way that new symbolic phrases consist of combinations of each note from the scale. In contrast to the main dataset we only recorded scales for four emotion classes, corresponding with each quadrant of the circumplex model. In addition to explaining the model to the vocalist, each quadrant had two key words which were angry/anxious, happy/exciting, relaxing/serene, sad/bored.

The data collection plan was based around common practice described by virtual instrument libraries^3^. For each emotion, 11 versions of a chromatic scale across an octave and a half were sung, 3 with very short notes, 3 with 500 ms, 3 with 1000 ms and 2 with 2000 ms duration. To allow the scales to contain all timbrel features for each emotion, we allowed for any dynamic variations and accents. The syllables themselves were also chosen for each scale by the vocalist.

## 4 Generative System Design

The system was designed with the primary goal of operating and responding to audio in real time on multiple embedded platforms. Future use cases will likely involve other computationally expensive systems, such as speech recognition and emotional interactions. In past work we have generated raw-audio for prosody (Savery et al., 2019b), however even after considerable refinement, and the use of multi-GPU systems, generation required 3 s of processing per 1 s of audio. With this in mind the initial design choice was to generate symbolic data using a version of the dataset converted to midi values, and not attempt to generate raw audio.

**FIGURE 2:**
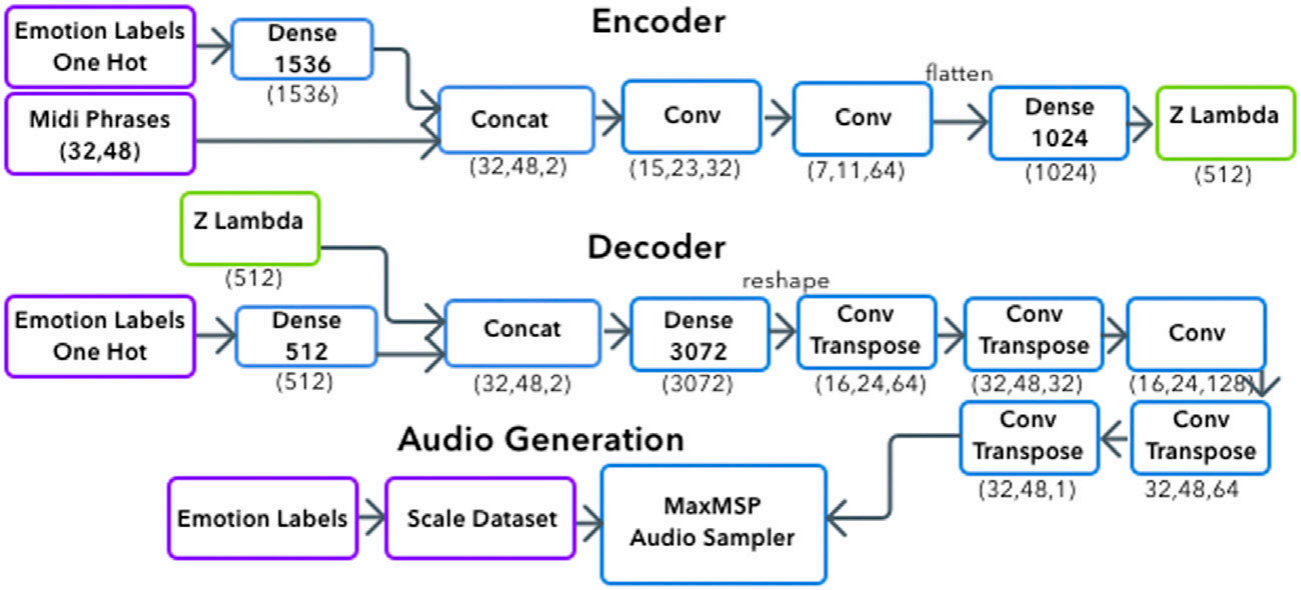
Generative System Overview.

**FIGURE 3:**
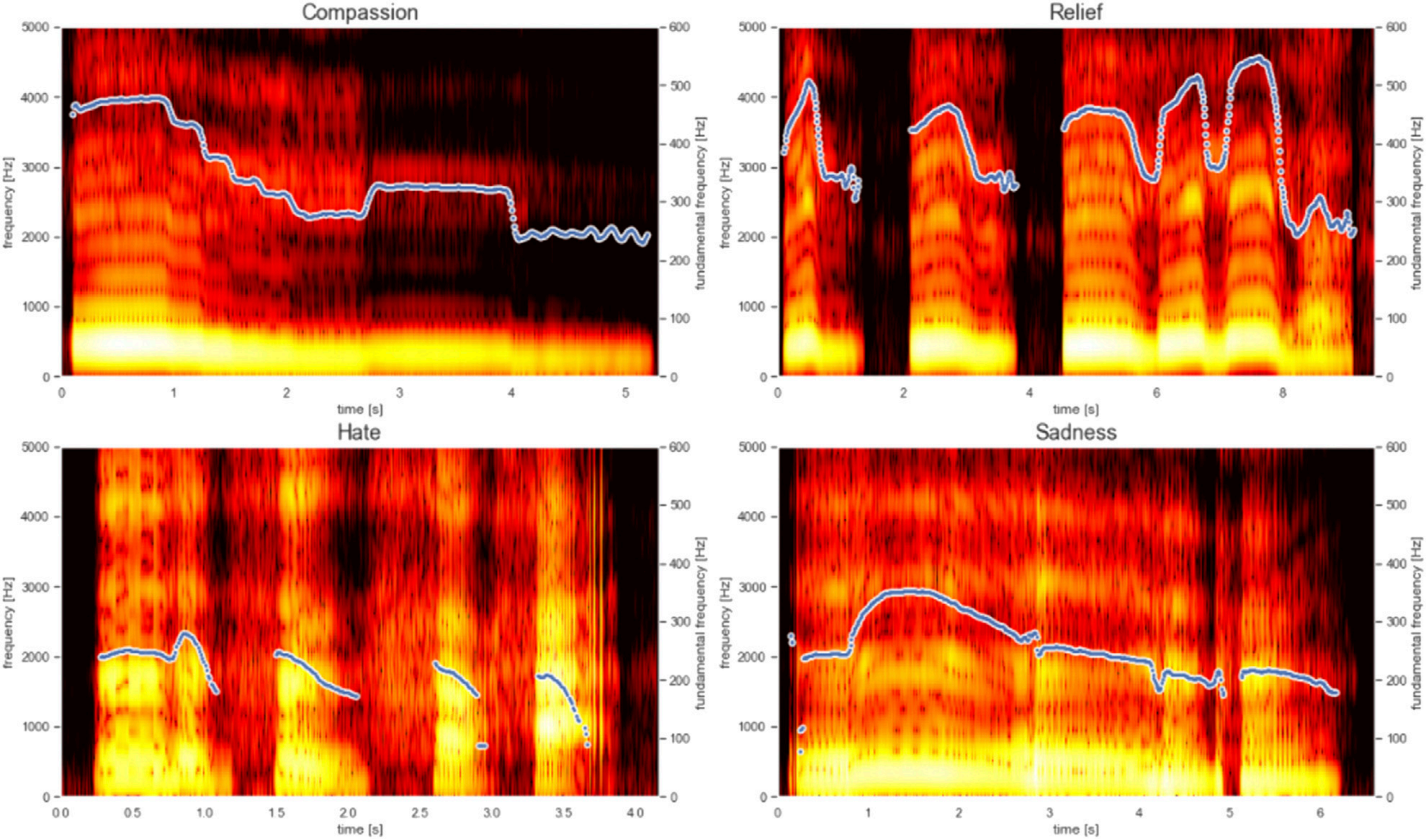
Spectogram of musical prosody phrases (blue line indicates pitch contour).

The symbolic generation of the system contains the pitch and rhythm of emotionally labeled melodies. Due to the process described in Section 3.3 the data also includes micro-timings. Symbolic data alone does not capture the range of emotion present in the dataset through timbre variations. By using the scale dataset described in Section 3.4 the generation process encapsulates symbolic information with tagged emotion, capturing timbre and phoneme information. Figure 2 shows an overview of the system. The system’s interface is written MaxMSP, allowing users to chose an emotion. This activates a python script which generates a midi file and returns it to MaxMSP. Figure 3 presents an example of the musical prosody phrases the systems is capturing, showing the contrasting pitch, rhythm and timbre for each emotion. Generated samples can be heard online.^4^

### 4.1 CC-VAE

#### 4.1.1 Data Representation

We maintain the same data structure as developed in our audio to midi process, using midi pitch values that are sampled every 10 milliseconds. We then convert each melody to a length of 1,536 samples, and zero pad shorter melodies. Versions of each phrase are then transposed up and down six semitones, to give 12 versions of each phrase, one in each key. The melody is then reshaped to be 32 by 48 samples. The emotion label for each melody is converted to a one-hot representation.

#### 4.1.2 Network Design

**FIGURE 4:**
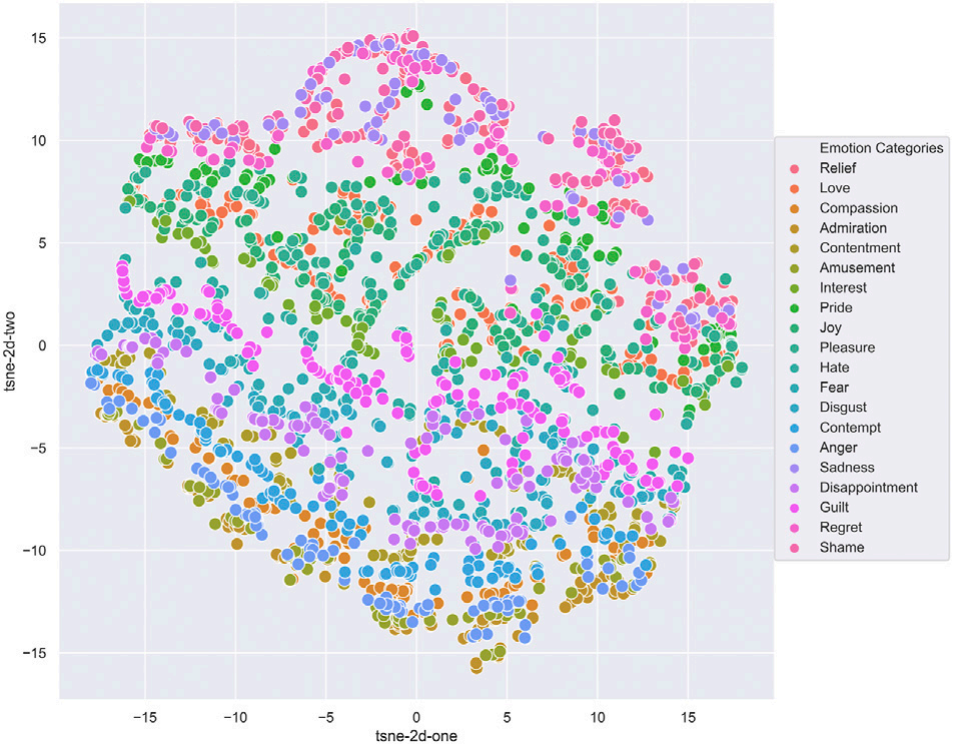
Vanilla VAE Latent Space, classifying Carter’s audio dataset.

We chose to use VAEs due to their recent success in sequence and music generation tasks, and because they allow for analysis of the latent space which can provide insight into how well the network has learned to represent the different emotions. VAEs can be used to generate new data by sampling and decoding from the latent space, allowing the system to learn features of the data in an unsupervised manner. Figure 4 shows the latent space after training a Vanilla VAE on our custom dataset, without emotion labels. This demonstrates the latent space is able to separate by emotion without conditioning.

Our Conditional VAE is based on the standard architecture proposed by Sohn et al. (Sohn et al., 2015). A Conditional Variational Encoder (CVAE) varies from a VAE by allowing an extra input to the encoder and decoder. We input a one-hot emotion label, allowing for sampling a specific emotion from the latent space. As is common practice for a VAE, we use Kullback-Leibler divergence in the loss function. Our latent space dimension is 512, which we arrived at after testing multiple variations.

We chose to use a Convolutional Network (ConvNet) within our CVAE for multiple reasons. Although ConvNets are much less common in symbolic music generation (Briot et al., 2017), they have been used for audio generation such as WaveNet (Oord et al., 2016) as well as some symbolic generations (Yang et al., 2017). While we experimented with Vanilla RNNs, LSTMs and GRUs as encoders and decoders we found they were very prone to overfitting when trained conditionally, likely due to our dataset size. Our architecture is presented in Figure 2.

### 4.2 Sample Player

The generated midi file is loaded into MaxMSP to be played by the sampler. The audio sampler plays back individual notes created during the recording of the scales. MaxMSP parses the midi file, assigning each note a midi channel. Channels are divided by emotion and note length. For example, happy is assigned to channels one to four, with channel one containing the shortest note and channel four the longest note; sad is assigned to channels five to eight with the shortest note assigned to channel five and the longest note assigned to channel 8. The audio sampler plays as a midi device, and can be played directly like any midi instrument.

### 4.3 Generation Evaluation

To evaluate the results, we first generated three phrases for each emotion. We then ran a survey using the same questions as the dataset validation described in Section 3.2, asking 39 new participants to select an emotion and intensity for each of the 60 total generated phrases. Participants encountered five listening tests during the survey, and we only used data from participants who answered all listening tests correctly. Figure 1 shows a comparison between the rose plots for each quadrant of the original dataset vs. the generated phrases.

We computed the mean and variance for each emotion, weighted by intensity, using the methods described in (Coyne et al., 2020), which rely on circular statistics. The results are shown in Table 1. The first columns show the percentage of all data points that were classified as an emotion in the correct quadrant. The next columns, showing average difference, were calculated by first finding the difference between each ground truth emotion’s angle and its weighted average reported angle, and then averaging that value over the emotions within each quadrant. It is worth noting that only three emotions in the dataset and two emotions in the generated data had weighted average angles outside the correct quadrant. The final units were converted from degrees to units of emotion (20 emotions in 360°). The last columns, showing variance, were calculated by finding the weighted variance for each emotion (converted to units of emotion), and then averaging for each quadrant.

**TABLE 1:** Results of emotion survey for dataset phrases compared with generated phrases. See *Generation Evaluation* for an explanation of the metrics.

Quadrant	% Correct Quadrant		Average Difference		Average Variance	
	Dataset	Generated	Dataset	Generated	Dataset	Generated
1	57.2	56.3	1.32	1.98	1.76	1.83
2	54.5	52.5	1.45	0.96	1.79	1.88
3	57.4	51.5	2.16	1.93	1.92	1.89
4	43.7	31.9	1.61	1.24	1.86	2.03

Our results show that the generated phrases performed similarly to the dataset in terms of emotion classification. While the percentage of phrases identified in the correct quadrant is slightly lower for the generated phrases, the average difference and variance have similar values. Visually, the rose plots show that participants were able to largely identify the correct quadrant, having the most difficulty with Quadrant 4 (relaxing/serene) for both our collected dataset and generations.

## 5 Experiment

**FIGURE 5:**
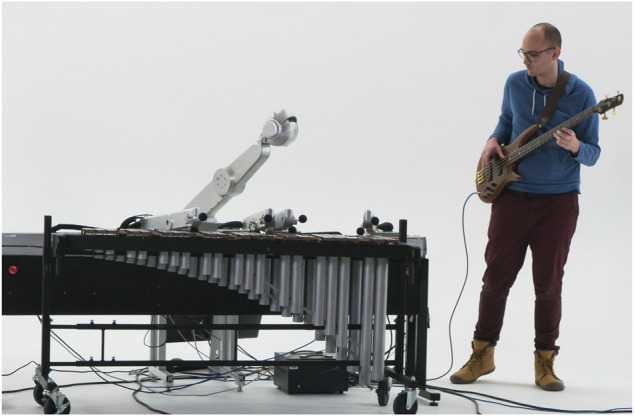
Shimon the robotic marimba player.

After creating the described prosody generation system we linked the system to our custom robotic platform Shimon. Shimon is a four-armed marimba playing robot that has been used for a wide range of musical tasks from improvisation (Hoffman and Weinberg, 2010) to film scores (Savery and Weinberg, 2018). Figure 5 shows Shimon improvising with a human performer. To visually show Shimon voicing the prosody we copied a previous implementation used to link Shimon’s gestures to human language for hip hop (Savery et al., 2020b).

For the experiment, we considered creativity through Boden’s framework for computational creativity (Boden, 2009). Boden considers creativity as a balance between novelty and coherence, with expressivity playing a significant role in the process. This concept draws on the notion that a completely random idea could be considered novel, yet would lack coherence. Boden’s framework was used to evaluate computational creativity in a number of previous works (Riedl and Young, 2010; Savery et al., 2020b).

We choose to compare musical prosody to a text-to-speech system for Shimon. Speech is very commonly used in robotics (Brooks et al., 2012; Niculescu et al., 2013) and is likely the primary form of audio interaction. Speech is often described as a way for replicating human to human communication (Crumpton and Bethel, 2016) and we believe would commonly be considered the default audio type for a robot such as Shimon.

Our experiment was designed to answer two research questions:

(1) Can emotional prosody improve a robot’s creative output, as measured through novelty, coherence and expressivity when compared to a text-to-speech system?
(2) Can emotional prosody alter the perception of animacy, anthropomorphism, likeability and intelligence for a creative robot compared to a text-to-speech system?

For these research questions we developed two exploratory hypothesis, extending the work of Moore (2017), where voices matching the mode of interaction will improve the interaction. For research question 1 we hypothesize that when communicating using emotion-driven prosody, Shimon will achieve higher ratings for novelty, and expressivity with a significant result, while coherence will not have significant difference. We hypothesize this will occur since prosody will increase the image of Shimon as creative agent, but not alter coherence. This aligns with our design goals of addressing the habitability gap and aiming for a robot that interacts in a manner that matches its performance. For research question 2 we hypothesize that there will be no difference in perception of animacy, and anthropomorphism, however prosody will achieve a significant result for higher likeability. We believe that the extra functionality implied by a text-to-speech system will enhance the perceived intelligence.

### 5.1 Experimental Design

We conducted the experiment as a between-group study, with one group watching robotic interactions with a text-to-speech system and the other with our generative prosody system. The study was set up as an online experiment with participants watching videos of a musician interacting with Shimon. For the text-to-speech we used Google API with a US female voice (en-US-Wavenet-E) (Oord et al., 2016). We chose the voice model as it is easily implemented in real time and a widely used system.

The musical interactions involved six clips of a human improvising four measures, followed by Shimon responding with a four-measure-long improvisation. The improvisation was over a groove at 83 beats per minute, resulting in the improvisation lasting for about 23 s. Each improvisation was followed by a seven-second gesture and response from Shimon, either using text-to-speech or prosody. Both the speech and prosody used three high valence-low arousal and three low valence-low arousal phrases. The prosody or text-to-speech was overdubbed after recording allowing us to use identical musical improvisations from the human and robot. For text-to-speech we used phrases that designed by the author based on past interactions in rehearsal between human participants.

The high valence-low arousal text included the three phrases:

- Great work. What you played really inspired me to play differently. Could you hear how we were able to build off each others music?
- That was fun, it was good playing with you. I really liked hearing the music you played on keyboard, it worked well with what I played.
- Thanks so much for playing here with me, I thought what you played was really good. Let’s keep playing together.

The low valence-low arousal text included the three phrases:

- Let’s try it again soon, the more we play together the more we will improve. I’m going to listen to you really carefully next time
- That was a really good start, I enjoyed the way we interacted together. We should keep trying to work on it and get better.
- Did you listen to what I played? Do you think it worked well with what you played? The more we practice the better we can get.

Participants first completed a consent form outlining the process, and then read brief instructions on the experiment process. After watching three of the clips they were asked to rate them based on Boden’s metrics, then repeated the process for the next three clips. Boden’s metrics were rated on a seven point sliding scale. Participants were explicitly asked to rate the musical improvisation from the robot for each metric. Clips were randomly ordered for each participant. Additionally, a seventh clip was added as an attention check, which included an additional video. In this video, instead of sound, participants were asked to type a word that was asked for at the end of the survey.

After watching each interaction, participants rated animacy, anthropomorphism, likeability and perceived intelligence using the Godspeed measure (Bartneck et al., 2009). Each metric contained four or five sub-questions, which were averaged to give an overall rating. To conclude the experiment, participants answered demographic questions and were given an open text response to comment on the robot or experiment.

We used Amazon Mechanical Turk (MTurk) to recruit participants who then completed the survey through Qualtrics. MTurk is a crowd-sourcing platform created by Amazon that allows individuals and businesses to hire users to complete surveys. Participants were paid $2.00 upon completion of the survey, which took around 10 min. We allowed only MTurk Masters to participate, and required a successful job rate of 90%. We also monitored time to complete overall, and time spent to complete each question. We recruited 106 initial participants, four of whom failed the attention check. An additional two participants were disqualified as they completed the survey in under 5 min. As participants failed the attention check a new spot was immediately opened allowing us to reach 100 participants. In total we included data from 50 participants who heard the text-to-speech system and 50 who heard the prosody system. The mean age of participants was 44, ranging from 25 to 72, with a standard deviation of 11. The majority of participants were based in the United States (89) with the remaining in India (11). We found no difference in comparisons of the results between each country. Considering the gender of each participant, 39 identified as female, 60 as male and one as non-binary.

### 5.2 Results

Our analysis was conducted with a Jupyter Notebook, running directly on the exported CSV from qualtrics. Libraries for analysis included NumPy, and SciPy.stats.

#### 5.2.1 Creativity

**FIGURE 6:**
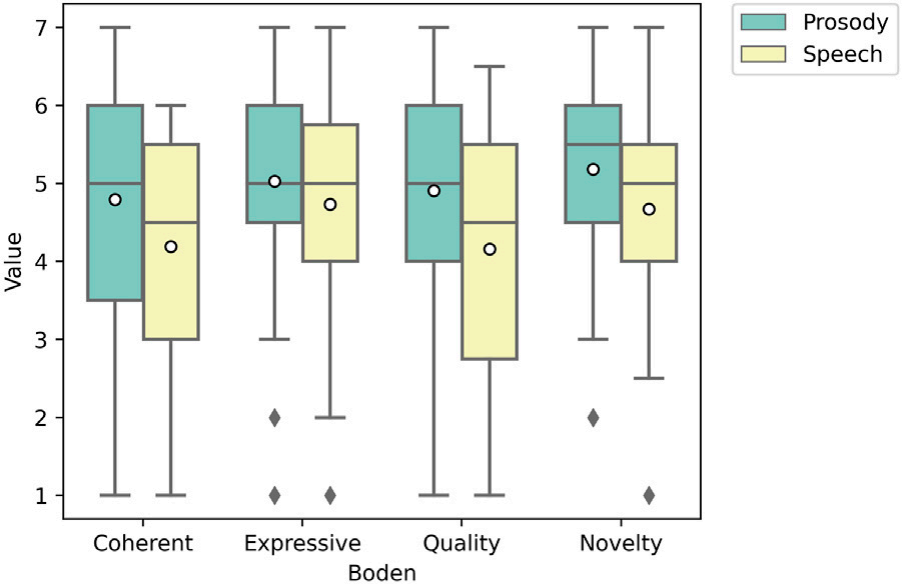
Box plot of Boden’s Creativity Metrics. Quality is significant, *p* = 0.014.

Prosody had a higher mean for coherence 4.80 (*std* = 1.31), novelty 5.18 (*std* = 1.30), and quality 4.95, (*std* = 1.68) compared to speech with the means 4.19 (*std* = 1.56), 4.64 (*std* = 1.24), and 4.14 (*std* = 1.37). Prosody had effect sizes of 0.40 for coherence, 0.43 for novelty, and 0.56 for quality indicating a medium size effect calculated using Cohen’s D. For expressivity prosody had an effect size of 0.25, indicating a small effect size. After conducting a pairwise *t*-test across categories were significant with the results, coherence (*p* = 0.041), novelty (*p* = 0.040), and quality (*p* = 0.014). After a Bonferroni-Holm correction for multiple comparisons, only quality remained significant with (*p* = 0.014) while coherence (*p* = 0.12) and novelty (*p* = 0.12) where no longer significant. For expressivity, prosody only had a slightly higher mean which was not significant (*p* > 0.05). Figure 6 shows a box plot of all Boden’s metrics.

#### 5.2.2 Godspeed

For the Godspeed metrics we first calculated Cronbach’s alpha for each question subset. This resulted in animacy (0.86), anthropomorphism (0.88), likeability (0.92), perceived intelligence (0.89). This shows high internal reliability across all metrics. Prosody had an effect size for each metric as animacy (0.16), anthropomorphism (0.08), likeability (0.85) and perceived intelligence (0.54), measured with Cohen’s D.

**FIGURE 7:**
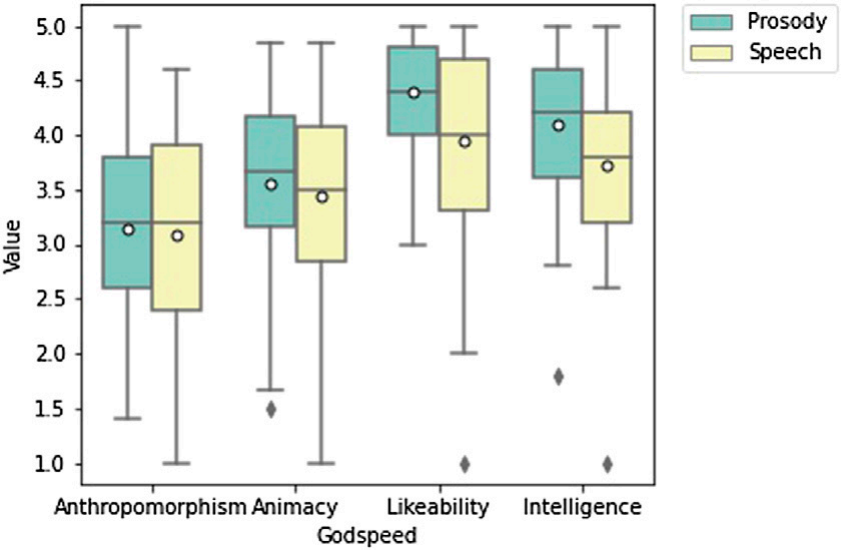
Box plot of Godspeed Metrics. Likeability and Intelligence are significant *p* = 0.011 and *p* = 0.042.

Prosody had a slightly higher mean for animacy 3.56 (*std* = 0.88) compared to speech 3.44 (*std* = 0.75). Prosody also had a slightly higher rating for anthropomorphism 3.14 (*std* = 0.99), compared to speech 3.08 (*std* = 0.885). After running a pairwise t-test neither animacy or anthropomorphism were significant. Prosody had a higher mean for likeability, 4.38 (*std* = 0.89) compared to 3.94 (*std* = 0.52) and showed a significant result (*p* = 0.002) in a pairwise *t*-test, which remained significant after a Bonferroni-Holm correction for multiple comparison (*p* = 0.011). For perceived intelligence, prosody 4.10 (*std* = 0.82) outperformed speech 3.72 (*std* = 0.70), with a significant result (*p* = 0.014) which remained significant after correction (*p* = 0.042). Figure 7 shows a box plot of all Godspeed metrics.

## 6 Discussion and Future Work

### 6.1 Research Question 1

Overall, our results indicated that the communication method outside of performance made a significant difference in participant ratings of creativity. The higher ratings for novelty and quality supported our hypothesis that prosody would outperform speech, however we did not expect coherence to improve with prosody as well. Surprisingly, we found no significant difference between voice type for expressivity and additionally expressivity only had a small effect size. This did not support our hypothesis as we had expected prosody to create the impression of a more expressive robot.

Further research is required to understand why the perception expressivity, as a creativity trait, did not change based on the voice used. One possible reason is that participants believed a robot that could use language was capable of a wide range of expression, much like the addition of prosody. Alternatively, expressivity is a feature that is not easily altered by the form of interaction post-performance.

The relation between each creativity rating cannot be easily simplified, and there is no correct answer to what rating a performance should receive for coherence or novelty. We expected that the prosody system would receive higher ratings for novelty, but not coherence. We believe that the higher ratings for coherence may have come from the system acting as a unified robot, with its communication functioning in the same manner as its performance.

### 6.2 Research Question 2

Our results for likeability matched our hypothesis that prosody would outperform speech. Perceived intelligence ratings however did not support our hypothesis as we had predicted language would be interpreted as having a higher intelligence. It was reasonable to assume that with text-to-speech and the ability to speak a language, Shimon would have been perceived as more intelligent. We found that the system with prosody was considered more intelligent, despite not communicating linguistically. This can be explained by the assumptions that moving towards the habitability gap will create a disjointed perception of the robot. A possible conclusion was that participants understood there was not a deep knowledge of language, whereas musical phrases implies a deeper musical intelligence.

### 6.3 Text Responses

We found no distinct variation in text responses between the speech and prosody group. Overall 92 participants chose to respond, with responses ranging from one sentence to four sentences. From the speech group only one participant mentioned the voice, writing “I enjoyed the robot, especially when she spoke to the pianist” (gender added by participant). In the prosody responses four participants mentioned the voice, but only in passing, such as the voice was “cute.” The vast majority of response rated the musical responses and generations, with the majority positive such as “I liked the robot and I like the robots music more than the humans,” and “Nice to listen to.” The negative comments tended to focus on the inability of robots in general to play music or be creative such as “It could play notes, but it lacked creativity.”

### 6.4 Generative Process

Our dataset used interpretation of emotions from one vocalist. While this had the benefit of consistency throughout phrases, in future work we intend to gather data from a larger number of musicians and to evaluate how well the model can generalize. We also plan to have other robots communicating through prosody using data from different vocalists.

We plan to further investigate timbre and its potential application to the generation process. We also intend to study which features of the phrases influenced participants’ choice in selecting an emotion. For example, exploring whether there is a difference in emotion classification accuracy for the melody of the generated phrases alone, in comparison with emotionally-sampled audio as we used here. Future work will also include more extensive studies using the generated prosody in human-robot interactions. This will take place between varying group sizes from one human and robot, to groups of humans and robots with different embedded personalities. We expect for emotional musical prosody to enable many future collaborations between human and robot. Our overall accuracy presented in Table 1 shows consistent results in the mid 50%. We believe this accuracy is acceptable for our current system, as the average variance and average difference are both close to two across all categories, implying that the primary errors in identification where small, such as mistaking love for admiration. For our experiment in particular we only used two quadrants, and were also able to choose only specific emotions that scored over 80% accuracy.

In both the original dataset and generated material participants had the lowest accuracy identifying the fourth quadrant emotions. Our results are not easily compared to other generative systems as the fourth quadrant emotions are rarely used in robotic studies Savery and Weinberg (2020). This is partly because common classification systems such as Ekman’s discrete classes do not include anything in the fourth quadrant. We also believe these emotions tend to be less easily displayed externally as they are low arousal and closer to neutral emotions. In future work we aim to consider methods to better develop the fourth quadrant emotions.

### 6.5 Limitations

We compared one text-to-speech system with one musical prosody system on one robotic platform. In future work we aim to compare further audio systems, to expand understandings of why different metrics showed significant results. It is possible that varying the speech used would alter the final ratings. Nevertheless, we believe that the range of metrics that did prove significant show that this is an important first step in understanding how communication between core creative tasks can shape the perception of a robot.

We were only able to compare two forms of communication in a the constrained scenario consisting of directly after a musical interaction. To restrict our experiment to two groups we did not compare prosody to moments where the robot did not interact at all. We believe that by its nature a robot such as Shimon is always interacting and its presence can alter humans actions (Hoffman et al., 2015), leading us to believe that no movement or audio is its own form of interaction. In future research we intend to analyze the impact of musical prosody compared to no interaction in a longer performance.

This study was conducted online through video, which comes with benefits and drawbacks. As we were running online we were able to gather many more participants than would have been possible in person. Similar HRI studies have shown no difference in online replication of certain studies (Woods et al., 2006), and we believe our method was constrained to a point that would be replicated in an in-person study. We did not include a manipulation check in our study, however our analysis of the text responses indicated that participants did not identify the independent variable between groups.

The range of participants included in the study also adds some limitations. Our primary goal was to understand how changes to a creative system would generalize across a broad population. We did not factor in concerns between cultural groups that may take place, such as between Japan and United States (Fraune et al., 2015), however our study did not find any significant variation between origin country. Additionally, our ability to generalize is restricted by only collecting participants on MTurk, who it has been shown do not always represent standard population samples, such as in the case of participants health status (Walters et al., 2018). Finally, our sample size of 106 participants was under the total that would be required to detect an effect size of 0.50 with 0.80 power at an alpha level of 0.05, which requires a sample size of 128.

## 7 Conclusion

The paper presents a new generative system for emotional musical prosody that is implemented in Shimon, a creative robot. We explore how a robot’s response outside of its key creative task—such as musical improvisation—alters the perception of the robot’s creativity, animacy, anthropomorphism, perceived intelligence, and likeability. Our research questions focus on how prosody compares to text-to-speech in a creative system for each of these HRI metrics.

We found that by addressing the habitability gap we were able to increase user ratings for the key creativity ratings; novelty and coherence, while maintaining ratings for expressivity across each implementation. Our results also indicated that by communicating in a form that relates to the robot’s core functionality, we can raise likeability and perceived intelligence, while not altering animacy or anthropomorphism. These findings clearly indicate the impact of developing interactions surrounding a creative performance, such as initial meetings and gaps between creative interaction.

Our results present wide ranging implications and future concepts for the development of creative robots. The importance of design outside primary tasks should not only be considered for creative robots, but across HRI. These findings indicate that embodiment and external design choices alter not only the impression of the robot, but the impression of its primary functions. We also believe this work indicates the importance of audio design, and the impact on perception that changes to audio alone can have on a system. By designing audio for the system task and not relying on default audio methods it is possible to drastically change the perception of a robotic system.

## Data Availability

The raw data supporting the conclusion of this article will be made available by the authors, without undue reservation.

## Footnotes

1
https://maryesthercarter.com/

2
www.richardsavery.com/prosodycvae

3
https://www.spitfireaudio.com/editorial/in-depth/grow-your-own-samples/

4
www.richardsavery.com/prosodycvae
